# The Impact of Glycosylation of Osteopontin on Urinary Stone Formation

**DOI:** 10.3390/ijms21010093

**Published:** 2019-12-21

**Authors:** Go Anan, Tohru Yoneyama, Daisuke Noro, Yuki Tobisawa, Shingo Hatakeyama, Mihoko Sutoh Yoneyama, Hayato Yamamoto, Atsushi Imai, Hiromichi Iwamura, Yuki Kohada, Jotaro Mikami, Jun Ito, Yasuhiro Kaiho, Takahiro Yoneyama, Yasuhiro Hashimoto, Makoto Sato, Chikara Ohyama

**Affiliations:** 1 Department of Urology, Hirosaki University Graduate School of Medicine, Hirosaki 036-8562, Japan; goanan@tohoku-mpu.ac.jp; 2 Department of Urology, Tohoku Medical and Pharmaceutical University, Sendai 983-8536, Japan; 3 Department of Advanced Transplant and Regenerative Medicine, Hirosaki University Graduate School of Medicine, Hirosaki 036-8562, Japan; 4 Department of Cancer Immunology and Cell Biology, Oyokyo Kidney Research Institute, Hirosaki, Aomori 036-8243, Japan

**Keywords:** osteopontin, aberrant glycosylation, urolithiasis biomarker

## Abstract

Osteopontin (OPN) is a matrix glycoprotein of urinary calculi. This study aims to identify the role of aberrant glycosylation of OPN in urolithiasis. We retrospectively measured urinary glycosylated OPN normalized by urinary full-length-OPN levels in 110 urolithiasis patients and 157 healthy volunteers and 21 patients were prospectively longitudinal follow-up during stone treatment. The urinary full-length-OPN levels were measured using enzyme-linked immunosorbent assay and glycosylated OPN was measured using a lectin array and lectin blotting. The assays were evaluated using the area under the receiver operating characteristics curve to discriminate stone forming urolithiasis patients. In the retrospective cohort, urinary Gal3C-S lectin reactive- (Gal3C-S-) OPN/full-length-OPN, was significantly higher in the stone forming urolithiasis patients than in the healthy volunteers (*p* < 0.0001), with good discrimination (AUC, 0.953), 90% sensitivity, and 92% specificity. The *Lycopersicon esculentum* lectin analysis of urinary full-length-OPN showed that urinary full-length-OPN in stone forming urolithiasis patients had a polyLacNAc structure that was not observed in healthy volunteers. In the prospective longitudinal follow-up study, 92.8% of the stone-free urolithiasis group had Gal3C-S-OPN/full-length-OPN levels below the cutoff value after ureteroscopic lithotripsy (URS), whereas 71.4% of the residual-stone urolithiasis group did not show decreased levels after URS. Therefore, Gal3C-S-OPN/full-length-OPN levels could be used as a urolithiasis biomarker.

## 1. Introduction

Urolithiasis is one of the most common diseases effecting worldwide populations. It frequently causes acute pain and occasionally adversely affects kidney function. It is a common urological problem, with a lifetime prevalence of approximately 10% in men and 6% in women and its prevalence has been increasing in many developed countries [1,2]. The recurrence rate of urolithiasis has been reported to be 30–40% in 5 years and 50% in 10 years [3,4]. Urolithiasis is a metabolic syndrome that appears relatively early. Proper prevention of urolithiasis could lead to future prevention of other metabolic syndrome diseases. Therefore, the prevention and early detection of urolithiasis is vital importance. 

Osteopontin (OPN) is one of the most important components in the calcium stone matrix [5,6]. It is a glycosylated phosphoprotein expressed in a variety of tissues in the body [7]. OPN is involved in many cell activities, such as adhesion, migration, proliferation, cancer metastasis, inflammatory cell infiltration, angiogenesis, and bone resorption [8]. However, the detailed mechanism of OPN-related urinary stone formation remains unknown. There are two conflicting hypotheses regarding the role of OPN in urinary stone formation: as an inhibitor [9,10] or as a promoter [11,12]. In OPN knockout mice, the calculus was sandy and small, with low amount of calculus formation [6]. In the kidney, while urinary stones were formed, OPN expression in distal tubular cells was increased [13]. Therefore, OPN is one of the necessary components of urinary stone formation. Although, several studies have reported correlations between OPN proteins and genes and stone formation [8,11,14,15], there are no studies regarding the correlation between OPN glycosylation and stone formation. This study aims to identify the aberrant glycosylation profile of urine OPN related to urolithiasis. We retrospectively measured urinary aberrant glycosylated OPN in urolithiasis patients and healthy volunteers by using a lectin array and evaluated the glycan structure of urinary OPN by lectin blotting analysis. Furthermore, to evaluate the clinical significance of urinary glycosylated OPN as a urolithiasis biomarker, we also conducted a prospective longitudinal follow-up of urinary glycosylated OPN normalized to urinary full-length OPN (uFL-OPN) levels during stone treatment by ureteroscopic lithotripsy (URS).

## 2. Results

### 2.1. Identification of Urolithiasis-Related Aberrant Glycosylation Profile of Urine OPN in Retrospective Cohort Study

**Figure 1:**
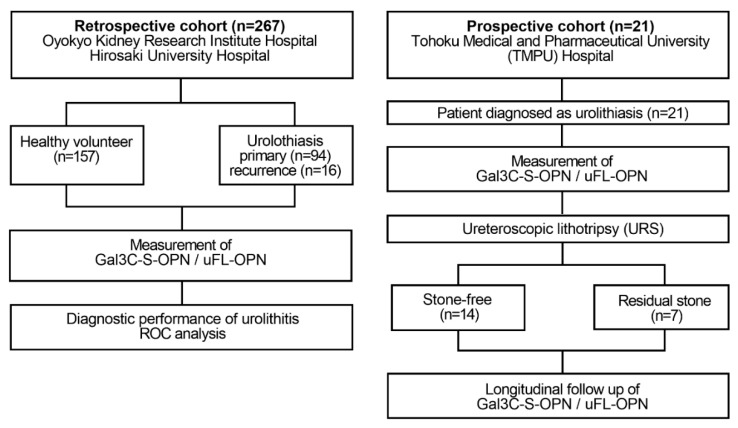
Flow diagram of the retrospective and prospective observational studies. The retrospective study enrolled 110 patients diagnosed with urinary calculi and 157 healthy volunteers who received health checks at Oyokyo Kidney Research Institute Hospital between June 2015 and August 2018. All urine samples were collected before stone treatment, and then the urine protein concentration was adjusted to 2 mg/mL followed by storage at −80 °C until use. A prospective cohort enrolled 21 patients with patients who were diagnosed with urinary calculi at Tohoku Medical and Pharmaceutical University Hospital in Sendai Japan between April 2018 and May 2019. Urine was collected prospectively in a patient diagnosed with urinary calculi during stone treatment. We divided the patients into two groups during stone treatment: the group without presence of stones after URS (stone-free group, *n* = 14) and the group with presence of stone after URS (residual-stone group, *n* = 7). As a diagnosis of urolithiasis, computed tomography was used for detecting the presence or absence of calculus. The definition of the presence of urolithiasis was based on CT imaging of a patient with a urinary calculus > 4 mm. The exclusion criteria were patients with renal atrophy, urinary catheter, and renal failure. Gal3C-S-OPN and uFL-OPN concentration were measured.

In the retrospective study, to identify the urolithiasis-related aberrant glycosylation profile of urine OPN, we investigated the mean fluorescence intensity (MFI) of lectin-reactive OPN normalized to uFL-OPN concentration in the healthy volunteers (HVs) and stone forming urolithiasis patients (Figure 1). 

**Figure 2:**
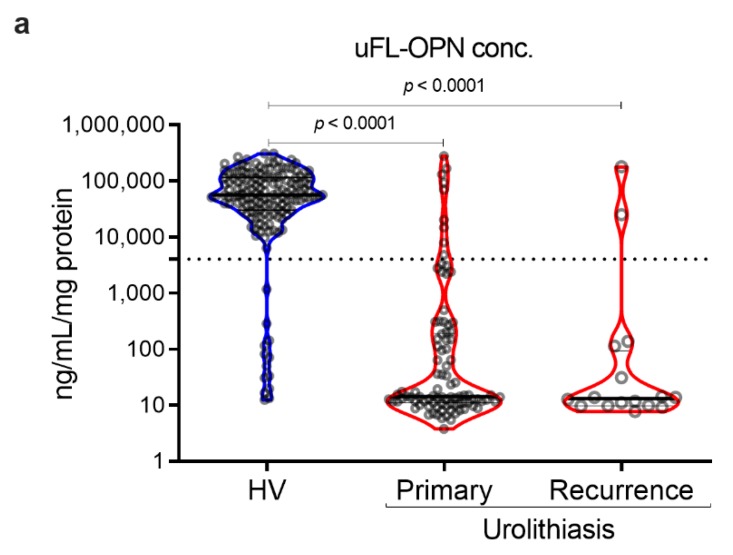

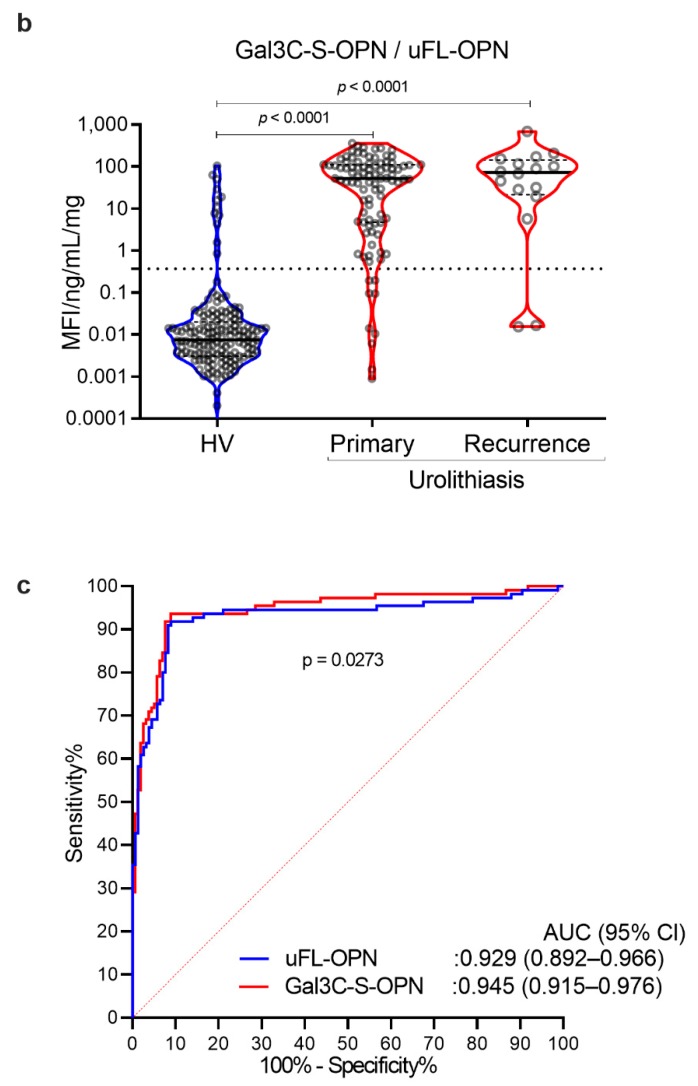
Detection of stone forming urolithiasis patients by Gal3C-S-OPN in the retrospective cohort. Urine levels and receiver operating characteristics (ROC) curve analysis of Gal3C-S-OPN normalized by uFL-OPN in patients diagnosed with or without urolithiasis. (**a**) Violin plot of uFL-OPN normalized by urine protein in healthy volunteers (HVs) and primary and recurrence urolithiasis patients. (**b**) Violin plot of Gal3C-S-OPN/uFL-OPN in HVs and primary and recurrence stone forming urolithiasis patients. Dashed black lines outline the interquartile range (IQR) of each test value. Solid black line represents the median of each test value. Multiple group differences were analyzed by using the Kruskal–Wallis test for non-normally-distributed models. (**c**) ROC curve of Gal3C-S-OPN and uFL-OPN for detecting urolithiasis patients.

As a result, the MFI of galectin 3 C-terminal-S (Gal3C-S) lectin, which recognized mucin-type *O*-glycan, -reactive OPN (Gal3C-S-OPN)/uFL-OPN level was significantly higher in primary stone forming urolithiasis patients [median, 52.3 MFI/ng/mL, interquartile range (IQR) 4.72–111.60] and recurrent stone forming urolithiasis patients (median, 72.0 MFI/ng/mL, IQR, 21.49–144.20) than that of HVs (median, 0.0074 MFI/ng/mL, IQR, 0.0030–0.0199]) (*p* < 0.0001, respectively) (Table 1, Figure 2). The area under the curve (AUC) of Gal3C-S-OPN/uFL-OPN for discriminating stone forming urolithiasis patients (AUC, 0.945; 95%Cl, 0.915–0.976) provided significantly better clinical performance than that of uFL-OPN (AUC, 0.929; 95%Cl, 0.892–0.945, *p* = 0.0273). At a preset 90% sensitivity with a cutoff value of 0.3706 MFI/ng/mL, the specificity of Gal3C-S-OPN/uFL-OPN to detect stone forming urolithiasis patients (92.41%) was higher than that of uFL-OPN (91.72%). 

Gal3C-S-OPN level was significantly higher in stone forming urolithiasis patients [median, 1118 MFI, interquartile range (IQR) 810–1543] than that of HVs (median, 515 MFI, IQR, 292–786]) (*p* < 0.001, respectively). We investigated sex-related differences. There was no statistical sex-related difference between Gal3C-S-OPN and Gal3C-S-OPN normalized to uFL-OPN in the healthy volunteers and stone forming urolithiasis patients. In the retrospective study, the stone component in 100 (91%) cases was calcium-containing stone and that in 10 (9%) cases was magnesium phosphate stone or uric acid stone (Supplementary Tables S1 and S2, Figures S1 and S2).

### 2.2. Immunoblotting and Lycopersicon Esculentum Lectin (LEL) Blotting Analysis

**Figure 3:**
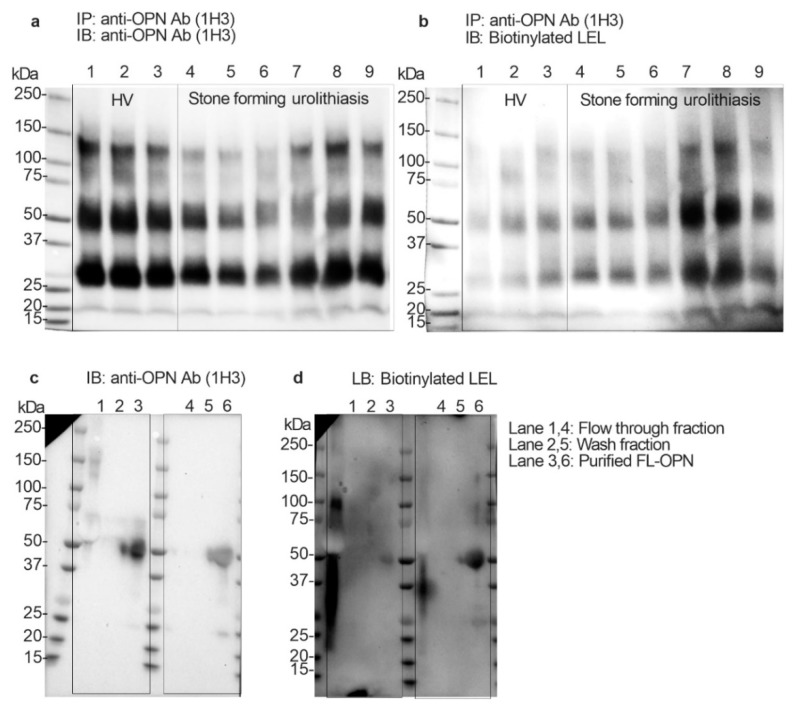
Immunoprecipitation and immunoblotting and *Lycopersicon esculentum* lectin (LEL) blotting analysis. (**a**) Immunoblotting analysis of concentrated urine (2 mg/mL protein) that was immunoprecipitated by anti-OPN Ab (1H3). Urine OPN was detected by anti-OPN Ab (1H3). (**b**) Lectin blotting analysis of concentrated urine (2 mg/mL protein) that was immunoprecipitated by anti-OPN Ab (1H3). Urine OPN was detected by LEL. Lanes 1 to 3: Healthy volunteers, Lanes 4 to 9: Urolithiasis patients with stones. Lanes 1 and 4: flow-through fraction of MonoPURE ProA spin column. Lanes 2 and 5: wash fraction of MonoPURE ProA spin column. Lanes 3 and 6 purified OPN fraction of MonoPURE ProA spin column. (**c**) Immunoblotting analysis of purified uFL-OPN from pooled urine of HVs or urolithiasis patients with stones. Urine OPN was detected by anti-OPN Ab (1H3). (**d**) Lectin blotting analysis of purified uFL-OPN from pooled urine of HVs or urolithiasis patients with stones. Urine OPN was detected by LEL. Lanes 1 and 4: flow-through fraction of MonoPURE ProA spin column, Lanes 2 and 5: wash fraction of MonoPURE ProA spin column, Lanes 3 and 6: eluted fraction of MonoPURE ProA spin column.

To determine whether the uFL-OPN had a poly-*N*-acetyllactosamine structure (polyLacNAc) or not, and whether its level of stone forming urolithiasis patients was significantly increased or not, we performed immunoblotting and *Lycopersicon esculentum* lectin (LEL) blotting analysis by using immunoprecipitated OPN from HVs and stone forming patients. Immunoblotting and lectin blotting analysis showed that the band intensity of uFL-OPN in the HVs was higher than that of the uFL-OPN in the stone forming patients, whereas the band intensity of LEL-reactive OPN clearly increased in stone forming urolithiasis patients (Figure 3a,b). We also performed immunoblotting and lectin blotting analysis of purified uFL-OPN from the pooled urine of urolithiasis patients or HVs. Immunoblotting analysis showed that the band intensity of purified uFL-OPN in HVs was higher than that of uFL-OPN in the stone forming patients, whereas LEL blotting analysis showed that the band intensity of LEL-reactive uFL-OPN clearly increased in the stone forming urolithiasis patients (Figure 3c,d). These results indicated that the uFL-OPN had a polyLacNAc structure and poly-*N*-acetyllactosamine glycosylated (polyLacNAcylated-) OPN level in urine was significantly increased in the stone forming patients.

### 2.3. Longitudinal Follow-Up of the Urinary Gal3C-S-OPN and uFL-OPN Level in Urolithiasis Patients during Stone Treatment

**Figure 4:**
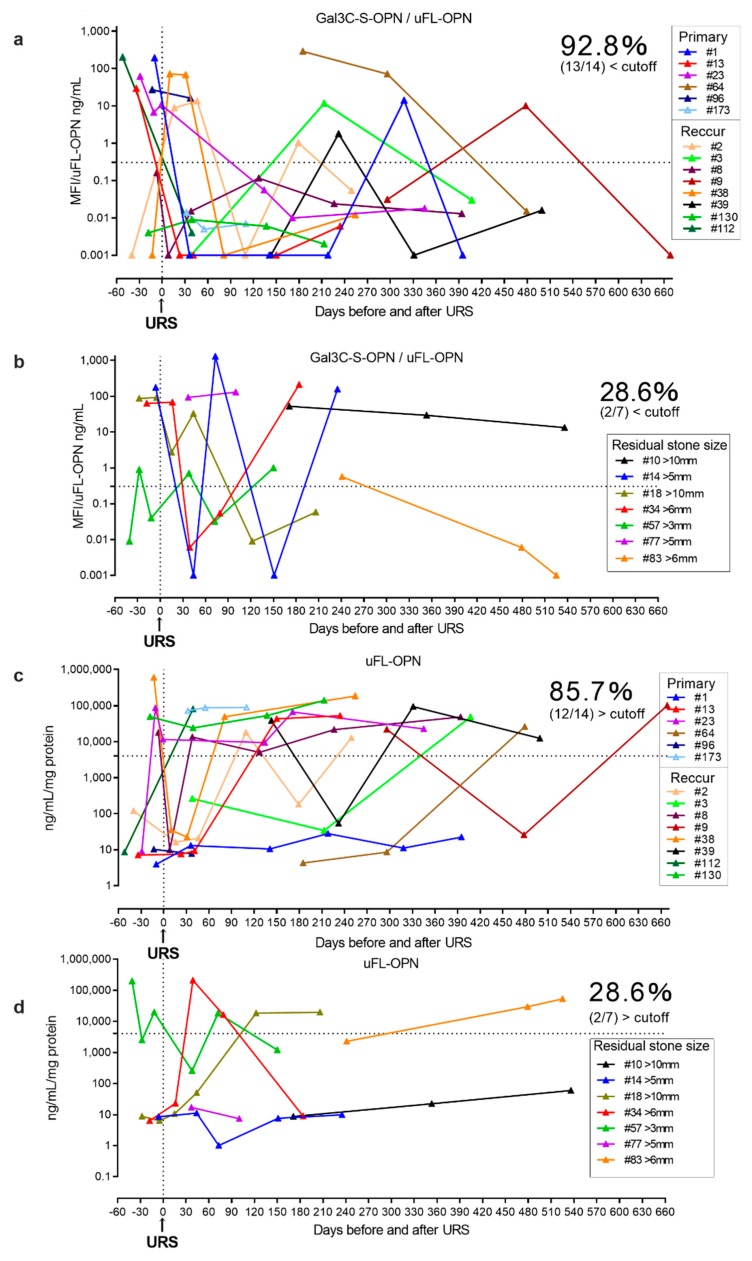
Longitudinal follow-up of Gal3C-S-OPN/uFL-OPN level in stone-free urolithiasis patients and residual-stone urolithiasis patients after URS. The urolithiasis-related aberrant glycosylation profile of OPN was analyzed by lectin array analysis. A 20-μL aliquot of concentrated urine was subjected to lectin chip analysis. (**a**) Longitudinal follow-up of Gal3C-S-OPN/uFL-OPN in stone-free urolithiasis patients after URS. (**b**) Longitudinal follow-up of Gal3C-S-OPN/uFL-OPN in residual-stone urolithiasis patients after URS. (**c**) Longitudinal follow-up of uFL-OPN in stone-free urolithiasis patients after URS. (**d**) Longitudinal follow-up of uFL-OPN in residual-stone urolithiasis patients after URS. Dashed black lines outline the cutoff value at 90% sensitivity as determined by a retrospective cohort.

Furthermore, we prospectively conducted a longitudinal follow-up of the urinary Gal3C-S-OPN/uFL-OPN level and uFL-OPN level in urolithiasis patients during stone treatment and compared the levels of Gal3C-S-OPN/uFL-OPN and uFL-OPN level between the residual stone group (*n* = 7) and stone-free group (*n* = 14) after URS (Table 2, Figure 4). The prospective longitudinal follow-up study showed a decrease in the level of Gal3C-S-OPN/uFL-OPN below the cutoff value (0.3706 MFI/ng/mL) in 92.8% (13/14) of the stone-free patients after URS, and 85.7% (12/14) of stone-free patients after URS showed a decrease in the level of uFL-OPN greater than the cutoff value (4073 ng/mL/mg). On the other hand, the level of Gal3C-S-OPN/uFL-OPN below the cutoff value (0.3706 MFI/ng/mL) was not decreased in 71.4% of the residual-stone urolithiasis patients (5/7) after URS, and the level of uFL-OPN greater than the cutoff value (4073 ng/mL/mg) was not decreased in 71.4% of the residual-stone urolithiasis patients (5/7) after URS. Therefore, Gal3C-S-OPN/uFL-OPN level was detected more than uFL-OPN in stone-free patients, which indicated that it could potentially be used as a urolithiasis biomarker.

In the prospective study, Gal3C-S-OPN level was higher but not statistically significant in residual-stone urolithiasis patients [median, 30 MFI, interquartile range (IQR) 0.001–176] than that of stone-free patients [median, 0.03 MFI, IQR, 0.001–12] (*p* = 0.08, respectively). In the prospective study, the stone component in 20 (95%) cases was calcium-containing stone and that in 1 (5%) case was uric acid stone (Supplementary Table S3).

## 3. Discussion

Several researchers have reported that urine OPN level-normalized creatinine was lower in stone forming urolithiasis patients than in HVs and also generated aberrant OPN forms (<40 kDa) [12,14,16,17]. OPN has important functions in cardiovascular diseases, cancer, diabetes and kidney stone diseases and in the process of inflammation, biomineralization, cell viability, and wound healing [18]. Use of urinary OPN level may be considered as a biochemical parameter to diagnose the risk of stone formation. Full-length osteopontin (FL-OPN) is a highly phosphorylated and glycosylated matricellular protein [19]. FL-OPN modulates cell function by interacting with cell-surface receptors, proteases, hormones, other bioeffector molecules, and structural matrix proteins, such as collagen [20]. The cleaved OPN by thrombin generates a functional fragment of *N*-terminal thrombin-cleaved OPN, which contains a cryptic binding site for integrin α9β1 and α4β1 that enhances the attachment of cleaved OPN to integrins [21]. Osteopontin is increased in the urine of lupus nephritis subjects, especially the cleaved form of osteopontin has been reported as a specific marker for the disease [22]. There have been some studies on OPN proteins and genes in urolithiasis [8,11,14,15], but there have been no studies on correlation between OPN glycosylation and stone formation. In the present study, we found that the uFL-OPN adjusted urine total protein was significantly lower in stone forming patients than in HVs (*p* < 0.001) and that urinary Gal3C-S-OPN/uFL-OPN level was significantly higher in stone forming urolithiasis patients than in HVs (*p* < 0.001).

Gal3C-S is a C-terminal domain of galectin-3 that reacts with the glycosylation containing galactose [23]. Gal-3C exhibited strong affinity to blood group A as well as blood group B antigens [24,25]. Gal-3C also displayed significant binding to both type 1 (Galβ1-3GlcNAc) and core 1 (Galβ1-3GalNAc) structures as well as to their 3′-*O*-sulfated forms, while binding to core 2 structure (Galβ1-3[GlcNAcβ1-6]GalNAc) was somewhat reduced [24,25]. Binding to these *O*-glycans was confirmed for asialoglycophorin [24,25]. In contrast, Gal-3C showed much weaker avidity to glycoproteins with galactosylated *N*-glycans [24,25]. These results suggest that Gal-3C might preferentially recognize mucin-type *O*-glycans rather than branched *N*-glycans [23]. According to above evidence, Gal-3C lectin react with mucin-type *O*-glycan on OPN.

Furthermore, in this study, we found that purified uFL-OPN in stone forming urolithiasis patients could be detected by LEL, whereas purified OPN in HVs could not be detected by LEL. The LEL has shown to react strongly to the polyLacNAc glycan structure [26]. Therefore, we consider there is a high probability of reacting with the Gal3C-S-OPN that is PolyLacNAc containing mucin-type *O*-glycosylated OPN. Mucin domain of human OPN contains five *O*-glycosylation sites (Thr^134^, Thr^138^, Thr^143^, Thr^147^, and Thr^152^) in the threonine/proline-rich region [27]. *O*-glycosylation influences the conformation, secretion, and proteolytic processing of the attached proteins [28,29,30,31,32], thereby affecting cellular functions, such as cell adhesion and migration. Kariya et al. reported that an OPN mutant with deletion of all five *O*-glycosylation sites (Δ*O*) increased its cell adhesion activity relative to that of wild type-OPN [27]. Change in the OPN glycosylation profile in stone forming urolithiasis patients is important as it may be related to OPN function. Two major *N*-glycan structures of osteopontin were also confirmed by mass spectrometry [33]. *N*-glycans of human osteopontin released by glycoamidase A were derivatized with 2-amino-pyridine. *N*-glycans are not essential sorting signals, suggesting that inner core carbohydrates and a proteinaceous signal mediate apical targeting of OPN [34].

The structure of polyLacNAc consists of a repeating structure that is *N*-acetyllactosamine (Galβ1,4GlcNAcβ1,3)n in which galactose (Gal) and N-acetylglucosamine (GlcNAc) are β1,4-linked. PolyLacNAc is a fundamental structure that is present in glycoproteins or glycosphingolipids. PolyLacNAc chain formation is considered to be important in the basic structure of the backbones of carbohydrate structures [35,36,37]. The structure of polyLacNAc itself functions as a ligand for endogenous lectins, such as galectin [24], and it has been suggested that the structure and length of polyLacNAc itself, which carries the sialyl Lewis antigen, is important in the grade of cancer and in intercellular interactions [38].

Although polyLacNAc is considered to be an important structure with various functions, there are no reports about correlation between polyLacNAcylated-OPN and urolithiasis. In this study, we demonstrated that polyLacNAcylated-OPN is related to the presence or absence of urolithiasis. The uFL-OPN could be detected by both polyLacNAc-reactive LEL and mucin type O-glycan reactive Gal3C-S lectin. Thus, it is thought that the polyLacNAc structure on mucin type *O*-glycosylated uFL-OPN in urinary stone forming urolithiasis patients could be reacted with endogenous lectins such as galectin 3 and might be have various direct or indirect biological functions. There are two possibilities to explain why the urinary polyLacNAcylated-OPN level of stone forming urolithiasis patients is higher than that of HVs. One possibility is that polyLacNAcylated-OPN might be a promoter of stone formation. The second possibility is that polyLacNAcylated-OPN might be an inhibitor of stone formation. Further molecular biological studies would clarify the biological significance of Gal3C-S-OPN on stone formation.

There were several limitations to this study. First, there were differences in the age and sex in the patient backgrounds between the two groups in the retrospective study. A future study should match the patient backgrounds and increase the number of cases. Second, in the prospective study, the number of cases was small. Third, in this study, we have not examined the urinary tract tissue; therefore, therefore it is unknown whether polyLacNAc is present in urinary tract tissue. Also, we tried mass spectrometry analysis of *N*- and *O*-glycan on uFL-OPN; however, a sufficient amount of OPN could not be purified from stone forming urine to analyze mass spectrometry. Also, we tried to extract FL-OPN by crushing the calculus; however, a sufficient amount of FL-OPN could not be purified. Also, plasma OPN concentration and urinary calcium oxalate measurement were not performed. Correlation polyLacNAcylated-OPN with urinary calcium oxalate was unknown. While the current study was small and retrospective, key observations were made concerning differences in OPN glycosylation states in stone former versus HV that were internally consistent across our data. To the best of our knowledge, this is the first study to identify aberrant glycosylation of OPN in urinary stone forming patients. We found that urinary Gal3C-S-OPN/uFL-OPN (AUC, 0.953) provided significantly better clinical performance for discriminating stone forming urolithiasis patients, with a sensitivity of 90% and a specificity of 92%. Furthermore, we prospectively conducted a longitudinal follow-up of the urinary Gal3C-S-OPN/uFL-OPN levels during stone treatment and showed that 92.8% of urolithiasis patients in the stone-free group had clear decreases in Gal3C-S-OPN/uFL-OPN levels after URS, whereas only 28.6% of the urolithiasis patients in the residual stone group after URS showed decreased Gal3C-S-OPN/uFL-OPN levels. This stone-free detection ratio (92.8%, 13/14) of Gal3C-S-OPN/uFL-OPN was better than that of uFL-OPN (85.7%, 12/14). These results suggest that Gal3C-S-OPN was increased in stone formation and decreased in the stone-free condition in urolithiasis patients. Therefore, Gal3C-S-OPN/uFL-OPN levels might be a biomarker of stone formation. Urolithiasis is a disease with a high rate of recurrence. Gal3C-S-OPN might be useful as a predictor of recurrence, and elucidation of the mechanism of urinary stone recurrence may lead to prevention.

## 4. Materials and Methods

### 4.1. Study Design and Assessments

A flow diagram of this observational study is shown in Figure 1. We evaluated the diagnostic performance of urinary glycosylated OPN normalized to uFL-OPN in determining stone forming urolithiasis patients. We measured urinary Gal3C-S reactive OPN normalized by urinary full-length-OPN levels (Gal3C-S-osteopontin/full-length-osteopontin). We retrospectively enrolled 110 patients diagnosed with urolithiasis and 157 HVs who received health checks at Oyokyo Kidney Research Institute Hospital between June 2015 and August 2018 (Table 1). A prospective cohort included 21 patients diagnosed with urolithiasis at Tohoku Medical and Pharmaceutical University Hospital between April 2018 and May 2019 (Table 2). Urine was collected prospectively in the patients diagnosed with urolithiasis during URS. The definition of the presence of urolithiasis was based on computed tomography (CT) imaging showing urinary calculus >4 mm. We classified the patients into two groups: the stone-free urolithiasis group after URS (*n* = 14) and the residual-stone urolithiasis group after URS (*n* = 7). The exclusion criteria were patients with renal atrophy, urinary catheter, and renal failure. All urine samples were collected before stone treatment and concentrated by using a VIVASPIN TURBO (MW 3000) (Sartorius, Gottingen, Germany), had their urine protein concentration adjusted to 2 mg/mL, and were then stored at −80 °C until measurement of urinary glycosylated OPN and uFL-OPN concentrations. We analyzed all the urine at one time. The retrospective study was approved by the ethics committee of Hirosaki University Graduate School of Medicine and Oyokyo Kidney Research Institute (“The Study about Carbohydrate Structure Change in Urological Disease”; approval number: 2019–001). The prospective study was registered with the Tohoku Medical and Pharmaceutical University Hospital Medical Research Registry in Japan (Protocol 2017-2-120) on 23 March 2018 and was registered with the University Hospital Medical Information Network Clinical Trials Registry in Japan (UMIN000035922). Written informed consent was obtained from all patients prior to participation in this study. The study was conducted in accordance with the principles of the Declaration of Helsinki and applicable clinical practice.

### 4.2. Quantification of uFL-OPN Concentration

The uFL-OPN concentration (ng/mL/mg total protein) was measured by using a human OPN enzyme-linked immunosorbent assay Kit (Immuno-Biological Laboratories Co., Ltd., Gunma, Japan) according to manufacturer’s instruction. Briefly, 100 μL of enzyme immunoassay buffer [1% bovine serum albumin (BSA), 0.05% Tween 20 in phosphate-buffered saline (PBST)] was added to an anti-human OPN rabbit IgG monocloncal antibody (O-17) precoated wells and then 100 μL of concentrated urine samples (2 mg/mL protein) or standards (0 to 320 ng/mL of human OPN) were added to each well. O-17 antibody reacted at part of the N-terminal of human OPN (IPVKQADSGSSEEKQ). The mixture was incubated for 1 h at 37 °C. The wells were then washed with PBST seven times. Then, 100 μL of (1×) horeseradish peroxidase (HRP) conjugated anti-human OPN mouse IgG monocloncal antibody Fab′ (10A16) in 1% BSA with PBST, was added to each well. The mixture was incubated for 30 min at 4 °C. 10A16 antibody reacted at part of the right side of the thrombin cleavage site of human OPN (KSKKFRRPDIQYPDATDE). The wells were then washed with wash buffer nine times. Then, 100 μL of tetramethyl benzidine solution was added to each well. The mixture was incubated for 30 min at room temperature in the dark. Then, 100 μL of Stop solution (1 N H_2_SO_4_) was added to each well. Measurements were made on a plate reader at 450 nm. The results shown are representative of three independent experiments.

### 4.3. Detection of Lectin-Reactive Glycosylated OPN in Urine by Using a Recombinant Lectin Array Chip

To identify the urolithiasis-related aberrant glycosylation profile of OPN, we performed lectin array analysis. Briefly, 20 μL of concentrated urine (2 mg/mL protein) was diluted with 80 μL of probing buffer (20 mM Tris-HCl, 150 mM NaCl, 0.1% Triton ×100, 1 mM CaCl_2_, and MnCl_2_). Diluted urine was added to the well of recombinant lectin array chip (Rexxam Co. Ltd., Osaka, Japan). After 70 min incubation, the diluted urine samples were aspirated and washed with probing buffer twice. Then, 100 μL of probing buffer containing 1 μg/mL biotinylated anti-OPN (1H3) antibody (Kerafast, Boston, MA, USA) was added to the well. 1H3 antibody reacted with any types of OPN. After 60 min of incubation, the antibody solution was aspirated and washed with probing buffer twice. Then, 100 μL of probing buffer containing 1 μg/mL of Cy3-labeled streptavidin (GE Healthcare, Buckinghamshire, UK) was added to the well. After 30 min incubation, the Cy3 solution was aspirated and washed with probing buffer twice. Then, the recombinant lectin array chip was scanned by using a Bio-REX Scan 200 evanescent fluorescence scanner (Rexxam Co. Ltd., Osaka, Japan). The mean fluorescence intensity (MFI) of lectin-reactive glycosylated OPN was normalized to uFL-OPN levels. The results shown are representative of three independent experiments. In this study, we examined urinary full length-OPN and not urinary cleaved form of OPN.

### 4.4. Immunoprecipitation and Immunoblotting and Lycopersicon Esculentum Lectin (LEL) Blotting Analysis

To confirm whether polyLacNAcylated-OPN was increased in stone forming urolithiasis patients, 500 μL of concentrated urine solution (2 mg/mL protein) from stone forming urolithiasis patients or HVs were mixed with 10 μL of anti-OPN antibody (1H3; Kerafast, Boston, MA, USA) for 3 h at 4 °C. Then, 100 μL of Protein G conjugated Dynabeads (ThermoFisher Scientific Laboratories, Waltham, MA, USA) was added to the urine-antibody mixture and incubated for 30 min at 4 °C. The OPN-antibody-protein G Dynabeads complex was collected by magnetic stand and washed with Tris-buffered saline with Tween20 (TBST) three times. Then, 20 μL of 3× sodium dodecyl sulfate (SDS)-denaturation buffer was added to the beads and incubated at 95 °C for 5 min. The eluents were resolved by SDS-polyacrylamide gel electrophoresis (PAGE) on a 4–20% gradient gel (Gellex International, Tokyo, Japan) and transferred onto a polyvinylidene fluoride membrane. The membrane was blocked with 5% BSA in TBST. Western blotting analysis was performed by using the anti-OPN antibody (1H3), which reacts with all forms of OPN, and a HRP-conjugated secondary antibody. Lectin blotting analysis was performed by using 5 μg/mL of biotinylated LEL in TBST, which reacts with the polyLacNAc glycan structure, and HRP-conjugated streptavidin. Signals were detected by using a Novex ECL Chemiluminescent Substrate Reagent Kit (ThermoFisher Scientific Laboratories, Runcorn, UK).

### 4.5. Purification of uFL-OPN from Pooled Urine of Urolithiasis Patients or Healthy Volunteers

To immunoprecipitate the uFL-OPN, 200 μg of anti-human OPN rabbit IgG (O-17; Immuno-Biological Laboratories Co., Ltd., Gunma, Japan) was added to the 8 mL of pooled urine from urolithiasis patients or HVs (*n* = 8, each) and incubated overnight at 4 °C. Then, the urine-antibody mixture was purified by using a MonoPURE ProA spin column (KikoTech Co., Ltd., Osaka, Japan) Kit according to the manufacturer’s instruction. Then, 500 μL of purified OPN-anti-OPN antibody mixture was concentrated to 20 μL by using Amicon^®^ Ultra centrifugal filter units 30 K (Millipore, Burlington, MA, USA). A 20-μL aliquot of the OPN-anti-OPN antibody mixture was resolved by SDS-PAGE on a 4–20% gradient gel. After electrophoresis, the gels were stained by using a negative staining method with EzStain Reverse (ATTO Corporation, Tokyo, Japan). A 50-kDa band containing uFL-OPN was cut from the gel and transferred into a 1.5-mL BioMasher tube (Takara-bio, Shiga, Japan), and 500 μL of Laemmli SDS-PAGE running buffer (25 mM Tris, 192 mM Glycine, 0.1% SDS) was added to remove the staining reagent. After 10 min of incubation, the above step was repeated once. Then, the supernatant was added to 200 μL of TBS buffer and gel pieces were homogenized by using a pestle and then incubated at room temperature for 1 h. After incubation, the homogenate was transferred to an ATTOPREP MF (ATTO Corporation) filtration tube and centrifuged (14,000× *g*) for 10 min at 4 °C. The filtered solution was collected as purified uFL-OPN solution from the urolithiasis patients or HVs. A total of 150 μg of purified uFL-OPN was subjected to LEL blotting analysis.

### 4.6. Statistical Analysis

Statistical analyses were performed by using Graphpad Prism 8 (GraphPad Inc., San Diego, CA, USA), XLSTAT-Biomed (Addinsoft, New York, NY, USA), and R software version 3.5.2 (R Foundation for Statistical Computing; available on: http//www.r-project.org/). For a non-normally distributed model, the Mann–Whitney *U*-test was used to analyze intergroup differences, and multiple group differences were analyzed by the Kruskal–Wallis test. The predictive accuracy was quantified as AUC of the receiver operating characteristics curve (ROC). In all analyses, a two-sided *p*-value of ≤ 0.05 was considered to be indicative of statistical significance.

## 5. Conclusions

In summary, this study showed that OPN concentration in urine was significantly lower in stone forming patients than in HVs. However, Gal3C-S-OPN was significantly increased in stone forming patients, which suggested its potential as a biomarker of urolithiasis formation.

## Figures and Tables

**Table 1:** Patients’ characteristics and results (retrospective study).

Group	Urolithiasis		Healthy	*p*-Value ^5^		
	Primary ^a^ (*n* = 94)	Recurrence ^b^ (*n* = 16)	Volunteers ^c^ (*n* = 157)	a vs. b	b vs. c	a vs. c
Sex (male/female)	55/39	6/10	57/100	0.3553	>0.9999	0.019
	median (IQR ^1^)	median (IQR ^1^)	median (IQR ^1^)			
Age (years)	67 (60–73)	67 (49–71)	46 (37–57)	0.9333	0.0003	<0.0001
uFL-OPN ^2^ (ng/mL/mg protein)	14.4 (11.1–191.1)	13.3 (9.8–93.3)	56,392 (30,271–115,517)	>0.9999	<0.0001	<0.0001
Gal3C-S-OPN ^3^ /uFL-OPN ^2^ (MFI ^4^/uFL-OPN ^2^)	52.3 (4.7–111.6)	72.0 (21.5–144.2)	0.007 (0.003–0.020)	>0.9999	<0.0001	<0.0001

^1^ IQR, Interquartile range; ^2^ uFL-OPN, Urinary full-length-osteopontin; ^3^ Gal3C-S-OPN, Gal3C-S lectin reactive osteopontin; ^4^ MFI, mean fluorescence intensity; ^5^ *p*-value, Kruskal–Wallis test. a, Primary stone forming urolithiasis patients; b, Recurrence stone forming urolithiasis patients; c, Healthy volunteers.

**Table 2:** Patients’ characteristics and results (prospective study).

Group	Urolithiasis after URS		*p*-Value ^5^
	Stone free (*n* = 14)	Residual stone (*n* = 7)	
Sex (male/female)	5/9	6/1	0.0635
	median (IQR ^1^)	median (IQR ^1^)	
Age (years)	61 (54–70)	74 (57–77)	0.3690
uFL-OPN ^2^ (ng/mL/mg protein)	120,003 (15.5–49,527)	35.6 (8.8–18,322)	0.0385
Gal3C-S-OPN ^3^ /uFL-OPN ^2^ (MFI ^4^/uFL-OPN ^2^)	0.02 (0.001–10.2)	1.87 (0.026–88.6)	0.0030
Follow-up period (day)	345 (213–407)	206 (150–525)	0.5815

^1^ IQR, Interquartile range; ^2^ uFL-OPN, Urinary full-length-osteopontin; ^3^ Gal3C-S-OPN, Gal3C-S lectin reactive osteopontin; ^4^ MFI, mean fluorescence intensity; ^5^ *p*-value, Mann–Whitney U-test.

## Supplementary Materials

Supplementary materials can be found at https://www.mdpi.com/1422-0067/21/1/93/s1.

## Abbreviations

OPN	Osteopontin
uFL-OPN	Urinary full-length osteopontin
URS	Ureteroscopic lithotripsy
MFI	Mean fluorescence intensity
HVs	Healthy volunteers
Gal3C-S-OPN	Gal3C-S reactive-osteopontin
polyLacNAcylated	Poly-*N*-acetyllactosamine glycosylated
IQR	Interquartile range
AUC	Area under the curve
LEL	*Lycopersicon esculentum* lectin
BSA	Bovine serum albumin
PBST	Phosphate-buffered saline
HRP	Horeseradish peroxidase
TBST	Tris-buffered saline with Tween20
SDS	Sodium dodecyl sulfate
PAGE	Polyacrylamide gel electrophoresis
ROC	Receiver operating characteristics curve
