# Supplementary material for: The Impact of Glycosylation of Osteopontin on Urinary Stone Formation
Source: Int J Mol Sci. 2019 Dec 21;21(1):93. doi: 10.3390/ijms21010093 (PMC6982307; doi:10.3390/ijms21010093)
Supplement: Supplementary file 1 [file ijms-21-00093-s001.pdf]

International Journal of Molecular Sciences

MDPI

Supplementary Table 1. The result of Gal3C-S-OPN between urolithiasis and healthy volunteers (retrospective study)

Group	Urolithiasis (*n* = 110)	Healthy volunteers (*n* = 157)	*p*-Value [5]
	median (IQR [1])	median (IQR [1])	
Gal3C-S-OPN [2] (MFI [3])	1118 (810–1543)	515 (292–786)	<0.001
uFL-OPN [4] (ng/mL/mg protein)	14 (10–151)	56392 (30270-115516)	<0.001
Gal3C-S-OPN [2] /uFL-OPN [4] (MFI [3]/uFL-OPN [4])	52 (5.2–113.0)	0.007 (0.003–0.020)	<0.001

[1] IQR, Interquartile range; [2] Gal3C-S-OPN, Gal3C-S lectin reactive osteopontin; [3] MFI, mean fluorescence intensity; [4] uFL-OPN, Urinary full-length-osteopontin; [5] *p*-Value, Mann–Whitney U-test.

Supplementary Table 2. Sex-related difference between Gal3C-S-OPN and Gal3C-S-OPN normalized to uFL-OPN (retrospective study)

Group	Urolithiasis		Healthy volunteers		*p*-Value [5]	
	Male [a] (*n* = 61)	Female [b] (*n* = 49)	Male [c] (*n* = 57)	Female [d] (*n* = 100)	a vs. b	c vs. d
	median (IQR [1])		median (IQR [1])			
Gal3C-S-OPN [2] (MFI [3])	1216 (888-1581)	972 (604-1529)	518 (301-854)	516 (278-781)	0.15	0.28
Gal3C-S-OPN [2] /uFL-OPN [4] (MFI [3]/uFL-OPN [4])	67 (9-120)	42 (4-103)	0.012 (0.003-0.042)	0.006 (0.002-0.014)	0.62	0.56

[1] IQR, Interquartile range; [2] Gal3C-S-OPN, Gal3C-S lectin reactive osteopontin; [3] MFI, mean fluorescence intensity; [4] uFL-OPN, Urinary full-length-osteopontin; [5] *p*-Value, Mann–Whitney U-test.

Supplementary Table 3. The result of Gal3C-S-OPN between urolithiasis and healthy volunteers (prospective study)

Group	Urolithiasis after URS		*p*-Value [4]
	Stone free (*n* = 14)	Residual stone (*n* = 7)	
	median (IQR [1])	median (IQR [1])	
Gal3C-S-OPN [2] (MFI [3])	0.03 (0.001–12)	30 (0.001–176)	0.08

[1] IQR, Interquartile range; [2] Gal3C-S-OPN, Gal3C-S lectin reactive osteopontin; [3] MFI, mean fluorescence intensity; [4] *p*-Value, Mann–Whitney U-test.

Supplementary Figure 1.

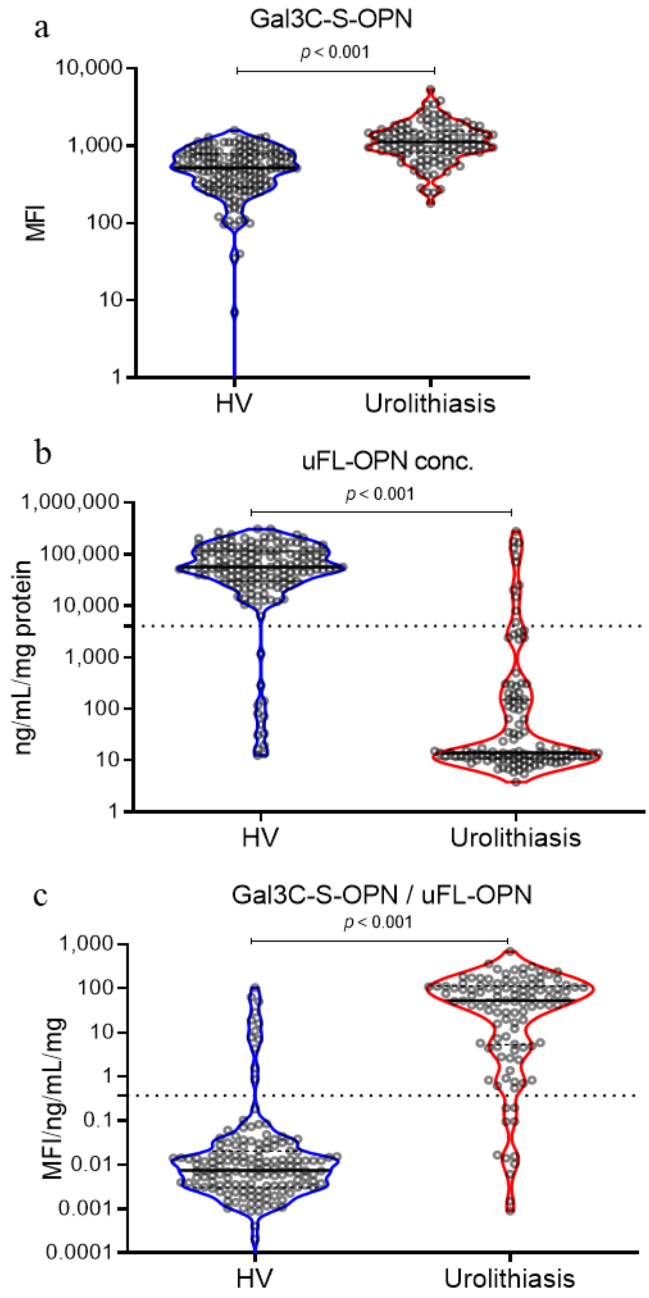

Supplementary Figure 1. Detection of stone forming urolithiasis patients by Gal3C-S-OPN in the retrospective cohort. **a**. Violin plot of Gal3C-S-OPN in healthy volunteers (HVs) and urolithiasis patients. **b**. Violin plot of uFL-OPN in HVs and urolithiasis patients. **c**. Violin plot of Gal3C-S-OPN normalized by uFL-OPN in HVs and urolithiasis patients. Dashed black lines outline the interquartile range (IQR) of each test value. Solid black line represents the median of each test value. Intergroup group differences were analyzed by using the Mann–Whitney U-test for non-normally-distributed models.

Supplementary Figure 2.

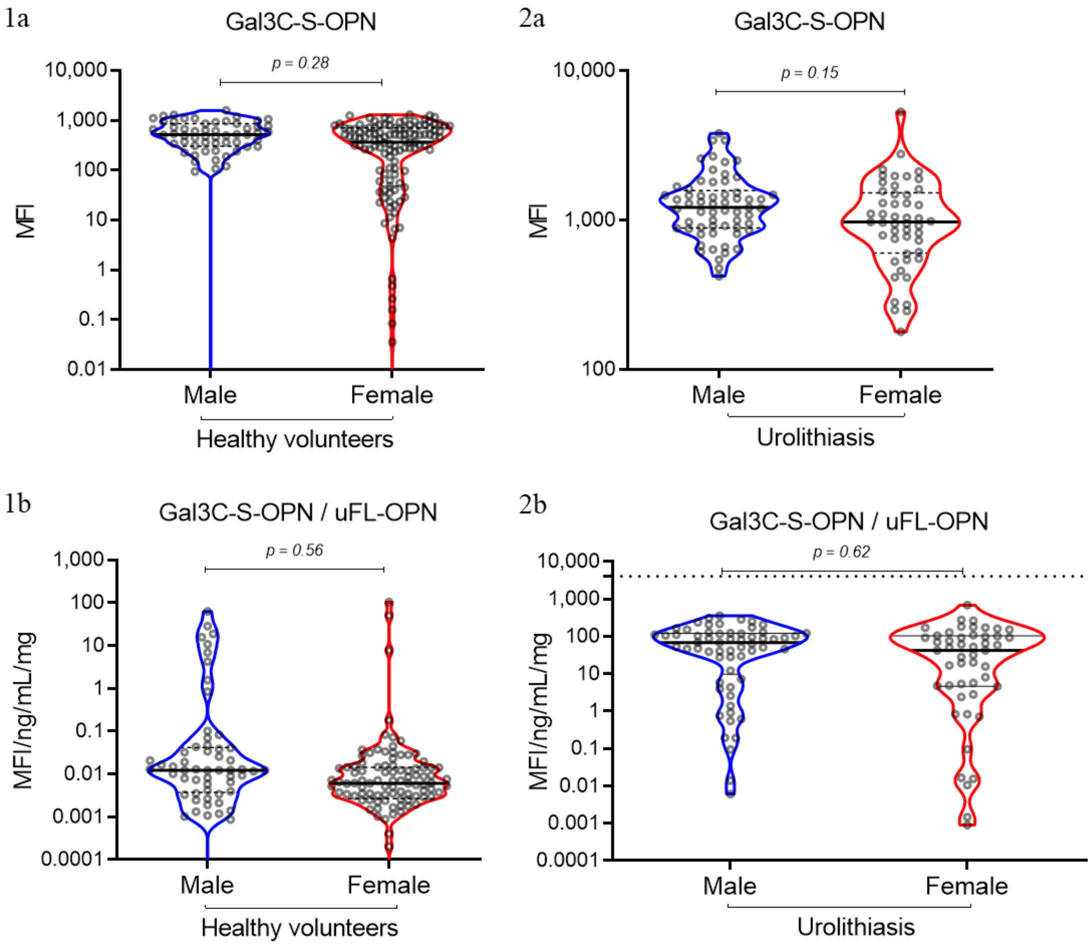

Supplementary Figure 2. Detection of sex-related differences by Gal3C-S-OPN in the retrospective cohort. 1**a**. Violin plot of Gal3C-S-OPN in male and female in the healthy volunteers. 1**b**. Violin plot of Gal3C-S-OPN normalized by uFL-OPN in male and female in the healthy volunteers. 2**a**. Violin plot of Gal3C-S-OPN in male and female in the urolithiasis patients. 2**b**. Violin plot of Gal3C-S-OPN normalized by uFL-OPN in male and female in the urolithiasis patients. Solid black line represents the median of each test value. Intergroup group differences were analyzed by using the Mann–Whitney U-test for non-normally-distributed models.
